# Ambulance quality and outcome measures for general non-conveyed populations (AQUA): A scoping review

**DOI:** 10.1371/journal.pone.0306341

**Published:** 2024-08-20

**Authors:** Erik Höglund, Carl Magnusson, Jakob Lederman, Douglas Spangler, Lilian Vloet, Remco Ebben

**Affiliations:** 1 Faculty of Medicine and Health, University Health Care Research Center, Örebro University, Örebro, Sweden; 2 Department of Molecular and Clinical Medicine, Institute of Medicine, University of Gothenburg, Gothenburg, Sweden; 3 Department of Clinical Science and Education, Södersjukhuset, Karolinska Institutet, Solna, Sweden; 4 Department of Surgical Sciences—Anesthesia and Intensive Care, Uppsala Center for Prehospital Research, Uppsala University, Uppsala, Sweden; 5 Radboud Institute for Health Sciences, Radboud University Medical Center, Nijmegen, The Netherlands; 6 Research Department of Emergency and Critical Care, HAN University of Applied Sciences, Nijmegen, The Netherlands; 7 Emergency Medical Service, Veiligheids- en Gezondheidsregio Gelderland-Midden, Arnhem, The Netherlands; Bach Mai Hospital, VIET NAM

## Abstract

**Background:**

An increasing number of patients receive ambulance care without being conveyed to a definitive care provider. This process has been described as complex, challenging, and lacking in guideline support by EMS clinicians. The use of quality- and outcome measures among non-conveyed patients is an understudied phenomenon.

**Aim:**

To identify current quality- and outcome measures for the general population of non-conveyed patients in order to describe major trends and knowledge gaps.

**Methods:**

A scoping review of peer-reviewed original articles was conducted to identify quality- and outcome measures for non-conveyance within emergency medical services. The Preferred Reporting Items for Systematic Reviews and Meta-Analyses extension for Scoping Reviews statement (PRISMA-ScR) was followed. The PROSPERO and OSF database were checked for pending reviews or protocols. PubMed, CINAHL, Scopus, Web of Science and the Cochrane Library database were searched for relevant articles. Searches were performed in November 2023.

**Results:**

Thirty-six studies fulfilled the inclusion criteria and were included in the review. Mortality was the most used outcome measure, reported in 24 (67%) of the articles. Emergency department attendance and hospital admission were the following most used outcome measures. Follow-up durations varied substantially between both measures and studies. Mortality rates were found to have the longest follow-up times, with a median follow-up duration a little bit over one week.

**Conclusions:**

This scoping review shows that studies report a wide range of quality and outcome measures in the ambulance setting to measure non-conveyance. Reported quality and outcome measures were also heterogeneous with regard to their follow-up timeframe. The variety of approaches to evaluate non-conveyance poses challenges for future research and quality improvement. A more uniform approach to reporting and measuring non-conveyance is needed to enable comparisons between contexts and formal meta-analysis.

## Introduction

Demand for Emergency Medical Service (EMS) care has increased over the past decade, driven by a patient population with increasingly complex health care needs, limited emergency care resources, and more frequent calls for primary care problems. [1–9]. With this increasing demand for ambulance care, an increasing number of patients receive ambulance care without being conveyed to hospital [10,11]. In this article, non-conveyance is defined as clinician-initiated, definitive on-scene care by an (ambulance) EMS clinician with any level of training and referral to any health care service other than conveyance by an ambulance to a hospital. The definition thus includes the practice of referring patients to the emergency department by alternate means (e.g. a private vehicle or taxi), to primary health care providers, and all other health care facilities and services. For general patient populations, the proportion of non-conveyed patients has been found to range from 3.7–93.7% [12]. The non-conveyed population is characterized by younger patients relative to conveyed patients. Common on-scene diagnoses for non-conveyed patients include abdomen and chest pain, breathing difficulties, trauma, low blood glucose levels, psychiatric problems, as well as a substantial number of patients with non-classifiable symptoms [13,14]. Non-conveyed patients have further been found to often have at least one abnormal vital sign [13–15]. This wide range of potential diagnoses, difficult-to-classify symptoms, abnormal vital signs, and plethora of alternate care pathways all contribute to a difficult decision-making process [13,15,16]. A significant proportion of ambulance patients seek health care following non-conveyance [15,17], though it is not clear what this entails in terms of patient safety [12]. This process has been experienced as complex, challenging, and lacking guideline support by EMS clinicians [12,18,19]. Patients have reported relatively high satisfaction with the care provided during non-conveyance, although it can evoke fear, shame, and a need for reassurance [20,21]. When not conveyed, and instead diagnosed and/or treated on-scene, patients can be referred to a wide range of health care services or left with no follow-up care. Although EMS clinician and patients have a positive attitude towards using alternate care pathways, the safety of the practice of referral to these alternatives remains unclear [22,23].

Historically, the safety of ambulance care has predominantly been measured using non-clinical, often time-related quality measures [24]. There is little to no evidence regarding the clinical benefits of evaluating EMS quality and performance using non-clinical quality measures [25]. The last two decades have seen the increasing use of clinical quality measures, which are most often applicable to specific and rare time-critical conditions (e.g. Out-of-hospital cardiac arrest). Thus, relatively few patients are impacted by their use [26]. Developing meaningful quality measures that holistically measure the quality of EMS care requires a clear definition of quality [27]. The concept of quality is highly contextual, which has resulted in a substantial variety of definitions of quality among clinicians, researchers, and society in general [28,29]. The use of quality measures among non-conveyed patients is an understudied phenomenon with a great variety of different outcome measures, making drawing generalizable conclusions difficult [12,30].

Therefore, to increase our knowledge regarding relevant outcome measures for the general non-conveyed patient population, we conducted a scoping review with the primary objective of identifying current quality and outcome measures for patients non-conveyed by EMS clinicians.

The aim of the study was to identify current quality and outcome measures for the general non-conveyance patient population in order to describe major trends and knowledge gaps.

## Methods

### Design

A scoping review was chosen since the research question was broad, and prior knowledge suggested that relatively few research papers described quality- or outcome measures for non-conveyed patients in the ambulance setting [31]. The scoping review is reported using the Preferred Reporting Items for Systematic Reviews and Meta-Analyses extension for Scoping Reviews statement (PRISMA-ScR) [32].

### Search strategy

The PROSPERO and OSF database were checked for pending reviews or protocols on the same topic, and none were identified. Systematic literature search strategies were developed for PubMed (National Center for Biotechnology Information, National Institutes of Health; Bethesda, Maryland USA), CINAHL (EBSCO Information Services; Ipswich, Massachusetts USA), Scopus (Elsevier, Amsterdam Netherlands), Web of Science (Clarivate, Philadelphia USA) and the Cochrane Library (The Cochrane Collaboration; Oxford, United Kingdom). All authors and information specialists at the Karolinska University library took part in the development of the search strategy. Searches were performed in November 2023. Full search strategies per database are described in (S1 File).

### Selection process

First, search queries for each database were downloaded and imported to BibDesk© v.1.8.11 where all hits were systematically deduplicated [33] and manually reviewed regarding duplication. Second, articles eligible for screening were imported to Rayyan [34]. Two pairs of independent researchers (EH/DS and CM/JL) each screened half of the identified records on title and abstract and decided on inclusion to the next step. The first step was conducted blinded between the two researchers. There were less than five percent conflicts of inclusion between all authors. Conflicts were resolved by consensus in full group discussions. Third, the articles eligible for inclusion were screened, blinded, in full text by the same two researchers. If articles were excluded in the full-text screening phase, the reasons for exclusion are reported in the flow diagram. Fourth, reference lists in all identified systematic reviews and scoping reviews were manually screened, first on title, then abstract and finally full-text articles in the same manner as in steps two and three.

**Inclusion criteria:** (1) Peer-reviewed research written in English, (2) All countries with no time constraints and including all study designs, (3) Research concerning non-conveyance within the ambulance service (e.g., not evaluations of emergency departments, primary health care, and dispatch centers.), (4) Articles reporting on the use or development of a quality- or outcome measures, (5) All provider levels were included, but had to be dispatched via the 9-1-1/1-1-2 system (e.g. not including community paramedicine interventions).

**Exclusion criteria:** (1) Articles reporting on clinical subgroups, for example; hypoglycemia and opioid overdose, (2) Articles clearly and only reporting on patient-initiated refusal of care, (3) Review articles.

All included articles were then divided between, and reviewed by one of the authors, who extracted all relevant information, including all identifiable quality and outcome measures. No evaluation of the quality of the included articles was performed since the main interest was the prevalence of reported quality and outcome measures in the literature rather than the findings of the studies.

### Analysis

The analysis was guided by the methodological framework described by Arksey and O’Malley (2005) [31]. The extracted measures were recorded in a Google Sheets document and then reviewed by the authors collaboratively. In this process, similar measures which had been written in different ways were combined (e.g., “EMS recontact” and “recontact with EMS” or “death” and “mortality”) and categorized into overarching types of measures. These data were then exported and processed using R (v 4.2.0) to generate descriptive statistics. The prevalence of each measure was described using absolute values and percentages. The follow-up time used in the studies for each measure was described in aggregate using box plots. The complete R code used to produce the analysis and generate tables and figures is available as (S2 File).

## Results

**Fig 1:**
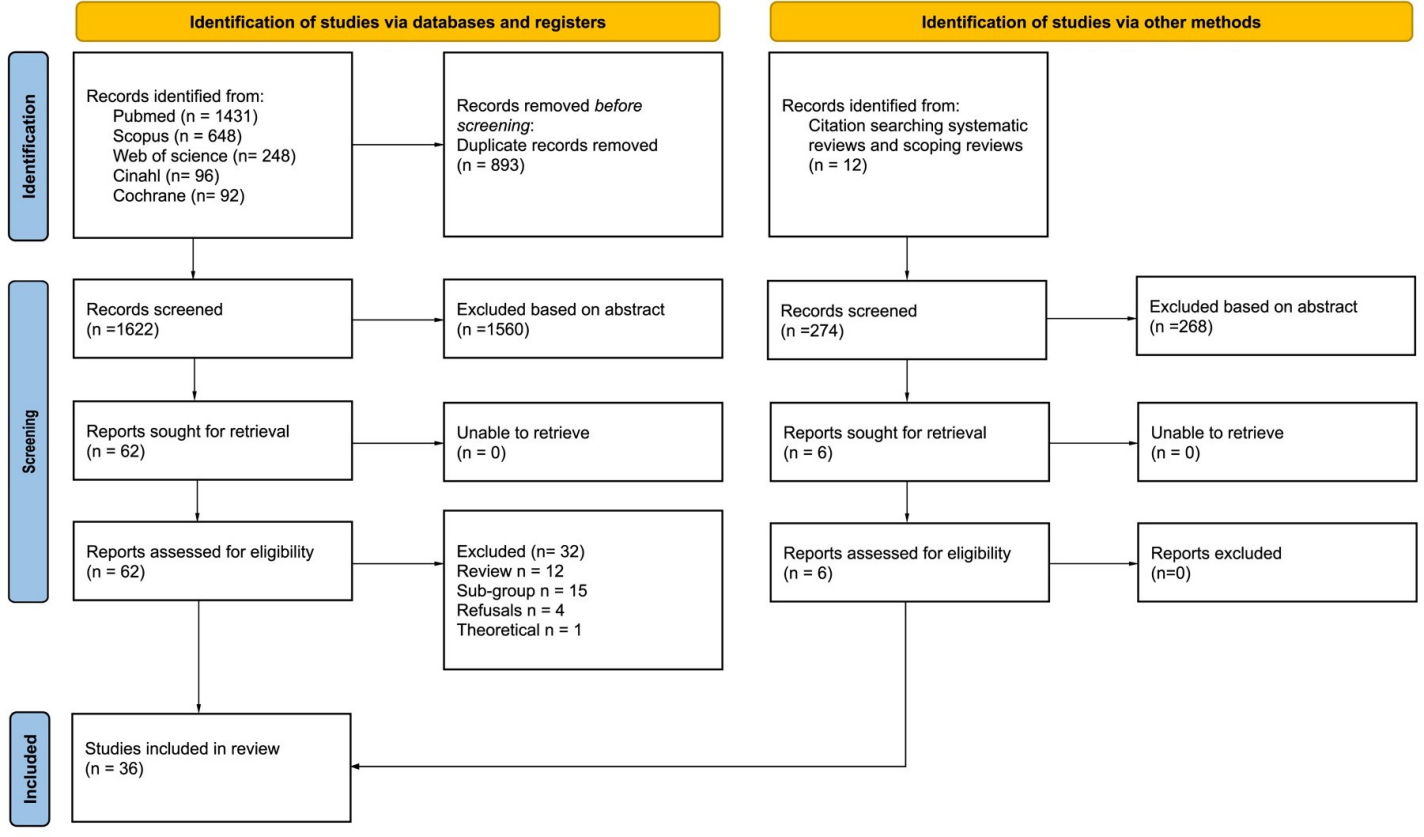
Flow diagram for the inclusion process.

From the database searches, 2,515 hits were identified. After deduplication, a total of 1,622 titles and abstracts were included for screening. In the first step, articles were included based on title and abstract. Of all screened articles, a total of 62 were retrieved to be read in full text, 32 of which were excluded. Of these, 15 articles were excluded due to the evaluation of sub-populations (e.g., hypoglycemia, falls, or opioid overdose). Twelve articles were not based on original research (i.e., review articles). Four articles evaluated only patient-initiated refusals, and one article concerned the evaluation of the theoretical ability of providers to predict patient outcomes. Thirty articles from the database searches fulfilled the inclusion criteria and were included in the review. The 12 excluded review articles were read in full text, and 274 references were extracted. These references were screened in the same way as the articles for the database searches. Of all review article references, six original articles were included after deduplication, abstract screening and full-text review. In total 36 studies fulfilled the inclusion criteria and were included in the review. The selection process is described in Fig 1.

Included articles were produced in the Sweden (7), USA (9), UK (5), Finland (4), Australia (4), Netherlands (1), Canada (3), Iran (1), and New Zealand (2). The ambulances were most commonly staffed by paramedics. A variety of study designs, Observational (n = 30), Mixed methods (n = 3), Qualitative (n = 1), Cluster-randomized (n = 1), Case-control (n = 1), and data collection approaches (Prospective (n = 14), Retrospective (n = 16) was used. Patients were of all ages, and various cut-off thresholds between 15–21 years were used to define adulthood. Fourteen studies included only adult patients, four studies included only children, one study included only elderly (>60 years) patients, and one study included only adult non-elderly patients (18–65 years). The remainder reported results for patients of all ages (Table 1).

**Table 1:** Description of included articles.

Title	Context	Study design	Population	Quality- or outcome measures used
Blodgett, (2020) [35]	UK (Paramedic)	Prospective observational	Age > = 17, n = 4540	Emergency department attendance, Hospital admission, Mortality, Emergency department interventions, Number of ward transfers
Bosson, (2023) [36]	USA (Paramedic)	Prospective observational	Age > = 18, n = 3330	Primary care, EMS recontact, Emergency department attendance, Mortality, Symptom progression, Any follow-up care after recommendation of no care, Patient satisfaction
Breeman, (2018) [37]	Netherlands (Nurse-based)	Prospective observational	Age > = 18, n = 1095	Primary care, Mobile care unit, EMS recontact, Emergency department attendance, Mortality, Any follow-up care after recommendation of no care, Referral to tertiary care, Patient satisfaction, Agreement about transport decision with physician
Carrigan, (2022) [38]	Canada (Paramedic)	Retrospective observational	All ages, n = 14072	EMS recontact, Mortality
Cooper, (2004) [39]	UK (Paramedic & ECP)	Mixed methods	All ages, n = 501	EMS conveyance rates
Coster, (2019) [40]	UK (EMT & Paramedic)	Retrospective observational	All ages, n = 42796	EMS recontact, Emergency department attendance, Hospital admission, Mortality
Forsell, (2021) [41]	Sweden (Nurse based)	Prospective observational	Age > = 18, n = 1048	Primary care visit, EMS recontact, Emergency department attendance, Hospital admission, Any follow-up care after recommendation of no care
Haines, (2006) [42]	USA (Paramedic)	Prospective observational	Age > = 21, n = 5336	Primary care visit, Emergency department attendance, Hospital admission, Mortality, Patient satisfaction
Heinonen, (2022) [43]	Finland (Mixed)	Retrospective observational	Age >16, n = 76233	Mortality
Höglund, (2022) [44]	Sweden (Nurse)	Prospective observational	All ages, n = 2691	Emergency department attendance, Hospital admission, ICU, Mortality
Jensen, (2013) [45]	Canada (Paramedic & ECP)	Retrospective observational	All ages, n = 238	EMS recontact
Kahalé, (2009) [46]	Canada (Paramedic)	Retrospective observational	Age < = 15, n = 345	Any follow-up care after recommendation of no care
Keene, (2015) [47]	Australia (Paramedic)	Qualitative	Age > = 18, n = 20	Referral to tertiary care, Primary care visit, Emergency department attendance
Knapp, (2009) [48]	USA (Paramedic)	Prospective observational	Age 18–65, n = 93	Emergency department attendance, Hospital admission, Adverse event
Langabeer, (2016) [49]	United States (EMT & Paramedic)	Case-control	All ages, n = 5570	EMS recontact, Mortality, EMS conveyance rates, Patient satisfaction
Larsson, (2017) [50]	Sweden (Nurse)	Prospective observational	Age > = 18, n = 3018	Hospital admission, Mortality, Any follow-up care after recommendation of no care
Laukkanen, (2022) [51]	Finland (Mixed)	Retrospective observational	All ages, n = 12530	Emergency department attendance, Hospital admission, ICU, Mortality, Physician consultation
Lederman, (2021) [52]	Sweden (Nurse)	Retrospective observational	Age >18, n = 17809	Emergency department attendance, Hospital admission, Mortality
Magnusson, (2016) [53]	Sweden (Nurse)	Retrospective observational	All ages, n = 529	Emergency department attendance, Hospital admission, Specific intervention
Magnusson, (2018) [54]	Sweden (Nurse)	Prospective observational	Age 0–15, n = 197	Emergency department attendance, Hospital admission, Mortality, Full triage, Specific intervention
Magnusson, (2020) [55]	Sweden (Nurse)	Prospective observational	Age > = 16, n = 6652	Emergency department attendance, Hospital admission, "Time sensitive condition", Adverse event
Mason, (2007) [56]	UK (Paramedic practitioners)	Cluster-randomized trial	Age >60, n = 394	EMS recontact, Emergency department attendance, Hospital admission, Mortality, Patient satisfaction, Quality of life, Specific intervention, Any follow-up care after recommendation of no care, Adverse event
Nehme, (2023) [57]	Australia (Paramedic)	Retrospective observational	Age < = 17, n = 62975	EMS recontact, Emergency department attendance, Hospital admission, Adverse event
Oulasvirta, (2019) [58]	Finland (EMT & Paramedic)	Retrospective observational	Age < = 15, n = 3579	Emergency department attendance, ICU, Mortality
Paulin, (2021) [59]	Finland (Nurse)	Prospective observational	All ages, n = 11861	Primary care visit, EMS recontact, Emergency department attendance, Hospital admission, Mortality
Peyravi, (2015) [60]	Iran (Paramedic)	Mixed methods	All ages, n = 3019	Mortality, Any follow-up care after recommendation of no care, Recovery
Pringle Jr, (2005) [61]	USA (Mixed)	Prospective observational	All ages, n = 310	Emergency department attendance, Hospital admission, Mortality, Any follow-up care after recommendation of no care
Schmidt, (2001) [62]	USA (EMT & Paramedic)	Prospective observational	All ages, n = 1433	Hospital admission, ICU, Specific intervention
Schmidt, (2006) [63]	USA (Paramedic)	Retrospective observational	All ages, n = 1581	Mortality
Snooks, (2004) [64]	UK (EMT & Paramedic)	Mixed methods	Age Not specified, n = 788	EMS recontact
Supples, (2022) [65]	USA (Mixed)	Retrospective observational	All ages, n = 3927	EMS recontact
Todd, (2021) [66]	New Zealand (EMT & Paramedic)	Retrospective observational	Age > = 15, n = 83171	Mortality, EMS conveyance rates, Any follow-up care after recommendation of no care, Recurrence of symptoms
Todd, (2021) [67]	New Zealand (EMT & Paramedic)	Retrospective observational	Age > = 15, n = 41406	EMS recontact, Mortality
Tohira, (2016) [68]	Australia (Paramedic & ECP)	Prospective observational	All ages, n = 67387	EMS recontact, Hospital admission, Mortality
Tohira, (2016) [69]	Australia (Paramedic)	Retrospective observational	All ages, n = 38293	EMS recontacts, Emergency department attendance, Hospital admission, Mortality
Zachariah, (1992) [70]	USA (Paramedic)	Retrospective observational	All ages, n = 158	Emergency department attendance, Hospital admission, ICU, Mortality

Several different quality and outcome measures were described in the included articles (Table 2). Most commonly reported quality or outcome measure involved an event or health care contact. Mortality was the most used measure, reported in 24 of 36 articles. Emergency department attendance and hospital admission were the following most used outcome measures.

**Table 2:** Description of identified quality and outcome measures.

Measure type	Measure name	Number of times each measure was reported	Most common timeframe or statistic (% of total)
Event/health care contact	Mortality	24 (67%)	1 week (29%)
Event/health care contact	Emergency department attendance	21 (58%)	3 days (38%)
Event/health care contact	Hospital admission	20 (56%)	3 days (40%)
Event/health care contact	EMS recontact	15 (42%)	2 days (40%)
Event/health care contact	Any follow-up care after recommendation of no care	9 (25%)	1 day (22%)
Event/health care contact	Primary care	6 (17%)	1 day (33%) / 3 days (33%)
Event/health care contact	ICU	5 (14%)	Not specified (60%)
Patient reported	Patient satisfaction	5 (14%)	Not specified (60%)
Event/health care contact	Specific intervention	4 (11%)	3 days (50%)
Event/health care contact	Adverse event	4 (11%)	1 month (25%) / 2 days (25%) / 48 hours (25%) / Not specified (25%)
Event/health care contact	EMS conveyance rates	3 (8%)	Immediate (33%) / 1 day (33%) / 2 days (33%)
Care duration	EMS	2 (6%)	Average 27min-35min (50%), Median 39min (50%)
Care duration	Hospital admission	2 (6%)	Median 3 days (50%) / not clearly stated (50%)
Event/health care contact	Referral to tertiary care	2 (6%)	Not specified (100%)
Care duration	Emergency department	1 (3%)	Not clearly stated (100%)
Time to care	Time from non-conveyance to Emergency department	1 (3%)	Average 43 min (100%)
Event/health care contact	"Time sensitive condition"	1 (3%)	3 days (100%)
Event/health care contact	ED Interventions	1 (3%)	1 month (100%)
Event/health care contact	Mobile care unit	1 (3%)	1 day (100%)
Event/health care contact	Number of ward transfers	1 (3%)	1 month (100%)
Event/health care contact	Recurrence of symptoms	1 (3%)	2 days (100%)
Expert evaluation	Agreement about transport decision with physician	1 (3%)	Not applicable (100%)
Guideline/protocol adherence	Full triage	1 (3%)	Not applicable (100%)
Guideline/protocol adherence	Physician consultation	1 (3%)	Not applicable (100%)
Patient reported	Quality of Life	1 (3%)	1 month (100%) / 3 days (100%)
Patient reported	Recovery	1 (3%)	Not specified (100%)
Patient reported	Symptom progression	1 (3%)	Not specified (100%)

**Fig 2:**
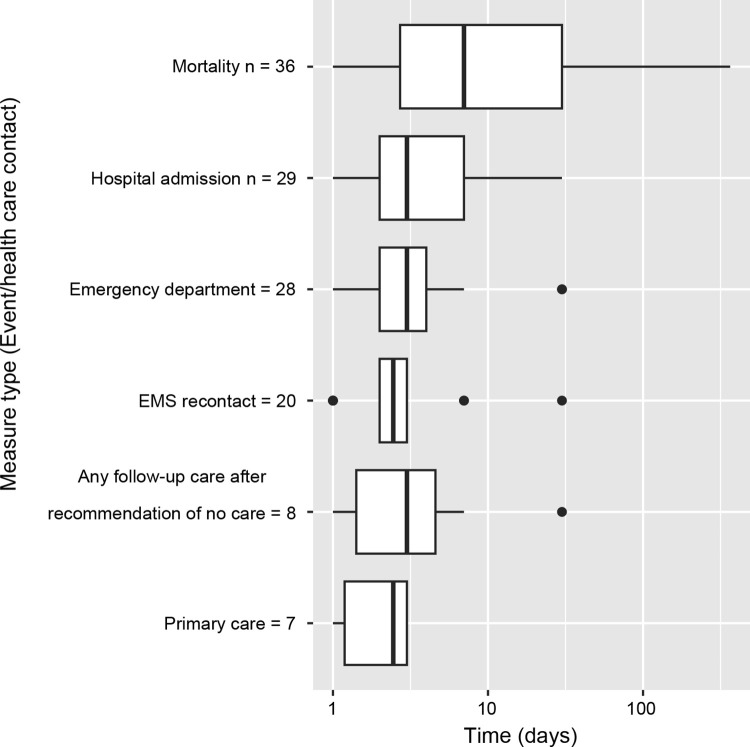
Box plot of follow-up durations of quality measures with >5 occurrences, log scaled. Note that n values are larger than in Table 2 due to studies reporting quality measures at multiple follow-up durations.

Follow-up durations varied substantially between both measures and studies, as described in Fig 2 below. Mortality rates were found to have the longest follow-up times, with a median follow-up duration of slightly over one week. Other measures had shorter follow-up durations, with all other measures with more than five occurrences in the literature having a median follow-up time of 72 hours or less.

## Discussion

This study found that patient mortality was the most commonly investigated quality or outcome measure following non-conveyance, reported in 24 (67%) of included studies. Measures of subsequent patient contact with the emergency department were found in 21 (58%) of all included studies, and hospital admission was reported in 20 (56%). The median follow-up duration for mortality was about one week, while emergency department visits and hospital admission measures were most commonly measured at three days. Outcome measures were often based on retrospectively gathered data. At the same time, only a few studies investigated patient-reported measures or measures based on expert review.

The interpretation of the identified measures varies substantially depending on the type of care pathway a patient is referred to following a non-conveyance decision (e.g., primary care or alternate transport to an emergency department). To increase the clinical relevance of the reported outcome measures concerning subsequent contacts, outcomes should be reported and stratified by non-conveyance destination.

The vast majority of the identified measures were based on all-cause data, and as argued by Ebben et al., health care re-contact rates are most relevant when they are related to the initial non-conveyance decision [12]. The validity and clinical relevance of the quality measures may thus be questionable. In an emergency department setting for instance, about 6 percent of repeated contacts within 72 hours were unrelated to the initial contact [71] while in a dispatching context, a 15% rate of unrelated contacts within 72 hours has been found [72]. Determining the relationship between two health care contacts can however be time-consuming, based on retrospective data, and such determinations have been found to have low interrater reliability [72]. The development of a standardized definition of whether two health care contacts are related could aid in promoting more valid and reproducible results. Many of the reported quality and outcome measures can only be obtained if data between different care providers (for instance, emergency dispatcher, primary care, EMS and emergency department) is linked, which is a barrier to data collection in some contexts [73].

We argue that the extent to which subsequent health care contacts, such as emergency department visits or hospital admissions, should be considered adverse events is questionable. The relevance of mortality over the long follow-up durations found in this study in measuring the quality of ambulance care is also open to question. The proportion of related contacts among subsequent health care contacts has been shown to decrease substantially over time [72]. Shorter-term mortality rates are arguably sufficient to capture situations where an adverse event might have occurred while minimizing sources of bias and random noise. Survival curves may offer a more nuanced alternative to reporting patient mortality rates over fixed durations [74–76]. It may furthermore be appropriate to exclude, or at least separately report, patients in end-of-life palliative care with short life expectancy from mortality statistics.

We found only one study evaluating the agreement between EMS clinicians and hospital clinicians, though additional studies evaluating such agreement rates were identified but excluded as they evaluated agreement only theoretically, e.g., whether a transported patient could have been left at home [77]. Performing expert reviews of medical records to identify triage errors has been found to be useful in similar contexts. Performing manual expert reviews of non-conveyance decisions determined to be “high risk” based on an automatically extracted quality measures may offer a fruitful approach to improving quality and limiting patient risks in the context of non-conveyance decisions in the EMS [72].

Not all articles stated who initiated the non-conveyance decision. Since the current study focused on describing quality and outcome measures relevant to clinician-initiated non-conveyance decisions, articles that clearly and only described patient-initiated refusals were excluded. Articles that did not clearly state who made the non-conveyance decision were still included to describe as many quality and outcome measures for the general non-conveyance population as possible.

This scoping review provides a high-level overview of how non-conveyance for the general non-conveyance population is measured and reported. How the described quality and outcome measures relate to patient safety remains unclear.

## Limitations

Scoping reviews have a significant limitation compared to systematic reviews in the generally broad nature of the research question, resulting most often in similarly broad and heterogeneous findings. This makes it more difficult to synthesize the findings comprehensively. Articles that fulfill the inclusion criteria could also be missed. Nonetheless, scoping reviews are appropriate for the aim of describing a body of research and identifying major patterns. The results of this review showed significant heterogeneity; therefore, results had to be abstracted to a high level. Although two to three authors reviewed each article and agreement between authors during the inclusion and exclusion process was high, no formal quality assessment of included articles was performed. This decision was made since the research question involved describing the quality and outcome measures used in the literature, rather than drawing conclusions regarding their findings.

## Conclusions

This scoping review shows that studies reported a wide range of measures in the ambulance setting to measure the quality- and outcomes of non-conveyance. The majority of included studies however included subsequent mortality, emergency department visits, and hospital admission. Studies were heterogeneous with regard to their follow-up timeframe, ranging from one day to one year. The most commonly reported outcome measures were in the form of mortality rates or subsequent contacts with the health care system following non-conveyance. There was a lack of patient-reported quality and outcome measures. The variety of approaches to evaluating non-conveyance poses challenges for future research.

A uniform approach to measuring and reporting non-conveyance is needed to enable comparisons between contexts and formal meta-analysis.

These findings can be used by researchers seeking to use standardized quality measures, outcome measures and follow-up durations in future studies to maximize the comparability of prehospital research. We furthermore identify a substantial reliance on outcome measures that can be easily extracted retrospectively from hospital records. Such measures are useful in terms of comparative evaluations and performing an initial selection for patients experiencing a potential adverse event. EMS organizations should consider various quality and outcome measures to capture multiple aspects of the phenomenon. The relevance of each quality and outcome measure needs further investigation. New quality and outcome measures may need to be developed to capture additional important aspects.

The current study revealed knowledge gaps related to uniformity in reporting strategies and the need for future studies to evaluate the clinical relevance of the used quality and outcome measures. The development of more sophisticated methods to more precisely identify adverse events resulting from non-conveyance should be sought. Our findings may be useful for EMS providers seeking to measure the complex phenomenon of non-conveyance.

## Supporting information

S1 File Search strategy for included databases. (DOCX)

S2 File R-code. (DOCX)

S3 File AQUA review data. (XLSX)

S4 File Aqua review analysis R script. (R)
