# Supplementary material for: Ambulance quality and outcome measures for general non-conveyed populations (AQUA): A scoping review
Source: PLoS One. 2024 Aug 20;19(8):e0306341. doi: 10.1371/journal.pone.0306341 (PMC11335110; doi:10.1371/journal.pone.0306341)
Supplement: S1 File — (DOCX) [file pone.0306341.s001.docx]

# Supporting Information

# S1 File. Search strategy for included databases.

**New PUBMED**

(Emergency Medical Services[Mesh:NoExp] OR Emergency Medical Service*[tiab] OR Medical Emergency Service*[tiab] OR EMS[tiab] OR emergency care[tiab] OR Transportation of patients[Mesh] OR ambulance*[tiab] OR emergency mobile unit*[tiab] OR mobile emergency unit*[tiab] OR Emergency Medical Technicians[mesh] OR Emergency Medical Technician*[tiab] OR EMT[tiab] OR paramedic*[tiab] OR emergency worker*[tiab] OR emergency provider*[tiab] OR emergency staff[tiab] OR emergency practitioner*[tiab] OR emergency nurs*[tiab] OR Rescue worker*[tiab] OR Rescue staff[tiab] OR Rescue nurs*[tiab] OR recovery worker*[tiab] OR recovery provider*[tiab] OR recovery staff[tiab] OR recovery practitioner*[tiab] OR recovery nurs*[tiab] OR EMS worker*[tiab] OR EMS provider*[tiab] OR EMS staff[tiab] OR EMS practitioner*[tiab] OR EMS nurs*[tiab]) AND ((Health Services Misuse[Mesh] OR transport need*[tiab] OR transportation need*[tiab] OR transport decision*[tiab] OR transportation decision*[tiab] OR non transport*[tiab] OR none transport*[tiab] OR non convey*[tiab] OR none convey*[tiab] OR discharge at the scene[tiab] OR treat and release[tiab] OR prehospital discharge[tiab]))

**Scopus**

#1 “Emergency Medical Service*” OR “Medical Emergency Service*” OR “EMS” OR “emergency care” OR “Transportation of patient*” OR “ambulance*” OR “emergency mobile unit*” OR “mobile emergency unit*” OR “Emergency Medical Technicians” OR “Emergency Medical Technician*” OR “EMT” OR “paramedic*” OR “emergency worker*” OR “emergency provider*” OR “emergency staff” OR “emergency practitioner*” OR “emergency nurs*” OR “Rescue worker*” OR “Rescue staff” OR “Rescue nurs*” OR “recovery worker*” OR “recovery provider*” OR “recovery staff” OR “recovery practitioner*” OR “recovery nurs*” OR “EMS worker*” OR “EMS provider*” OR “EMS staff” OR “EMS practitioner*” OR “EMS nurs*”

#2 (“Health Services Misuse” OR “transport need*” OR “transportation need*” OR “transport decision*” OR “transportation decision*” OR “non transport*” OR “none transport*” OR “non convey*” OR “none convey*” OR “discharge at the scene” OR “treat and release” OR “prehospital discharge”)

**Web of science**

#1 TS=(“Emergency Medical Service*” OR “Medical Emergency Service*” OR “EMS” OR “emergency care” OR “Transportation of patient*” OR “ambulance*” OR “emergency mobile unit*” OR “mobile emergency unit*” OR “Emergency Medical Technicians” OR “Emergency Medical Technician*” OR “EMT” OR “paramedic*” OR “emergency worker*” OR “emergency provider*” OR “emergency staff” OR “emergency practitioner*” OR “emergency nurs*” OR “Rescue worker*” OR “Rescue staff” OR “Rescue nurs*” OR “recovery worker*” OR “recovery provider*” OR “recovery staff” OR “recovery practitioner*” OR “recovery nurs*” OR “EMS worker*” OR “EMS provider*” OR “EMS staff” OR “EMS practitioner*” OR “EMS nurs*” )

#2 TS= (“Health Services Misuse” OR “transport need*” OR “transportation need*” OR “transport decision*” OR “transportation decision*” OR “non transport*” OR “none transport*” OR “non convey*” OR “none convey*” OR “discharge at the scene” OR “treat and release” OR “prehospital discharge”)

**Cinahl**

#1 (“Emergency Medical Service*” OR “Medical Emergency Service*” OR “EMS” OR “emergency care” OR “Transportation of patient*” OR “ambulance*” OR “emergency mobile unit*” OR “mobile emergency unit*” OR “Emergency Medical Technicians” OR “Emergency Medical Technician*” OR “EMT” OR “paramedic*” OR “emergency worker*” OR “emergency provider*” OR “emergency staff” OR “emergency practitioner*” OR “emergency nurs*” OR “Rescue worker*” OR “Rescue staff” OR “Rescue nurs*” OR “recovery worker*” OR “recovery provider*” OR “recovery staff” OR “recovery practitioner*” OR “recovery nurs*” OR “EMS worker*” OR “EMS provider*” OR “EMS staff” OR “EMS practitioner*” OR “EMS nurs*” )

#2 (“Health Services Misuse” OR “transport need*” OR “transportation need*” OR “transport decision*” OR “transportation decision*” OR “non transport*” OR “none transport*” OR “non convey*” OR “none convey*” OR “discharge at the scene” OR “treat and release” OR “prehospital discharge”)

**Cochrane**

Manual search on Prehospital , emergency medical services
