# Supplementary material for: Ambulance quality and outcome measures for general non-conveyed populations (AQUA): A scoping review
Source: PLoS One. 2024 Aug 20;19(8):e0306341. doi: 10.1371/journal.pone.0306341 (PMC11335110; doi:10.1371/journal.pone.0306341)
Supplement: S2 File — (DOCX) [file pone.0306341.s002.docx]

# Supporting Information

# S2 File. R-code.

# time_map <- c("Not specified" = NA,

# "Not applicable" = NA,

# "Other (note)" = NA,

# "Immediate" = 0,

# "1 day" = 24,

# "3 days" = 72,

# "1 week" = 168,

# "2 days" = 48,

# "1 month" = 720,

# "48 hours" = 48,

# "1 year" = 8760,

# "4 days" = 96,

# "10 month" = 7200,

# "<30 days" = 720)

# articles <- read_excel("AQUA_review.xlsx",

# sheet = "review_articles") %>%

# filter(Excl != 1,

# !is.na(`Author, year`),

# `Author, year` != "Laukkanen, 2021")

# measures <- read_excel("AQUA_review.xlsx",sheet = "review_measures") %>%

# filter(is.na(excl),

# !is.na(`Author, year`),

# `Author, year` != "Laukkanen, 2021") %>%

# mutate(Hours = as.numeric(plyr::revalue(`Time frame`,time_map)))

# t1 <- articles %>%

# left_join(select(measures,`Author, year`,`Measure name`),by ="Author, year") %>%

# group_by(`Author, year`) %>%

# summarize(`Measures used` = paste(unique(`Measure name`),collapse=", ")) %>%

# left_join(articles, by = "Author, year") %>%

# mutate(`Sample size` = as.numeric(`Sample size`),

# Title = paste(`Author, year`," - ",Title),

# Context = paste0(Country," (",`Provider level`,")"),

# Population = ifelse(`Age group`=="All",

# paste0(`Age group`," ages, n = ",`Sample size`),

# paste0("Age ",`Age group`,", n = ",`Sample size`))) %>%

# select(Title,Context,`Study design`,Population,`Measures used`)

# t2 <- measures %>%

# filter(`Author, year` %in% articles$`Author, year`) %>%

# group_by(`Measure type`,`Measure name`,`Time frame`) %>%

# summarise(`Author, year`,

# n_occur = length(unique(`Author, year`)),

# `Time frame`,

# related = paste(unique(Relation),collapse = ", ")) %>%

# group_by(`Measure type`,`Measure name`) %>%

# mutate(n_tot = length(unique(`Author, year`)),

# time_pct = n_occur/n_tot) %>%

# filter(time_pct == max(time_pct)) %>%

# summarise(`Number of ocurrences` = max(n_tot),

# `Most commom timeframe (% of total)` = paste0(unique(`Time frame`)," (",round(unique(time_pct)*100),"%)",collapse = " / ")) %>%

# ungroup() %>%

# arrange(desc(`Number of ocurrences`))

# (f1 <- measures %>%

# group_by(`Measure name`) %>%

# mutate(n=n(),

# `Measure name` = ifelse(`Measure name` == "Any follow-up care after reccomendation of no care",

# "Any follow-up care after\nreccomendation of no care",

# `Measure name`),

# `Measure name` = paste0(`Measure name`,", n = ",n)) %>%

# filter(n>5) %>%

#

# ggplot(aes(x=Hours,

# y=fct_reorder(`Measure name`,n))) +

# #geom_point() +

# geom_boxplot() +

# theme_minimal()+

# theme(panel.grid.minor = element_blank()) +

# labs(y = "Measure name") +

# scale_x_log10(breaks = c(0,24,48,72,168,720,8760)))

# write.csv(t1,"t1.csv",row.names = F)

# write.csv(t2,"t2.csv",row.names = F)
